# Predictive model of bulk drag coefficient for a nature-based structure exposed to currents

**DOI:** 10.1038/s41598-021-83035-0

**Published:** 2021-02-10

**Authors:** Alejandra Gijón Mancheño, Wiljan Jansen, Johan C. Winterwerp, Wim S. J. Uijttewaal

**Affiliations:** Faculty of Civil Engineering and Geosciences, Delft University of Technology, Stevinweg 1, 2628 CN Delft, The Netherlands

**Keywords:** Ecology, Environmental sciences, Hydrology, Ocean sciences, Engineering

## Abstract

Mangrove vegetation provides natural protection against coastal hazards like flooding and erosion. In spite of their economic and societal value, mangrove forests have experienced a worldwide decline due to human activities. Bamboo structures, formed by poles driven into the soil, are being used to create a sheltered environment for mangrove restoration. The lack of design rules for the structures has led to mixed success rates in their implementation. Improving future designs requires a better understanding of how the bamboo poles affect waves and currents. Currents cause drag forces on the poles, which depend on flow acceleration through the elements (blockage), and the distance from wakes of upstream cylinders (sheltering). We developed a model that predicts the bulk drag coefficient of dense arrays of emergent cylinders in a current, including blockage, sheltering and a balance between turbulence production and dissipation. The model could reproduce measured bulk drag coefficients from the literature within a deviation of 20%. The model also showed that anisotropic structures with small spanwise spacing and large streamwise separation maximize the bulk drag coefficient, and the energy dissipation per pole. The application of the model can guide the design of future mangrove restoration efforts.

## Introduction

**Figure 1:**
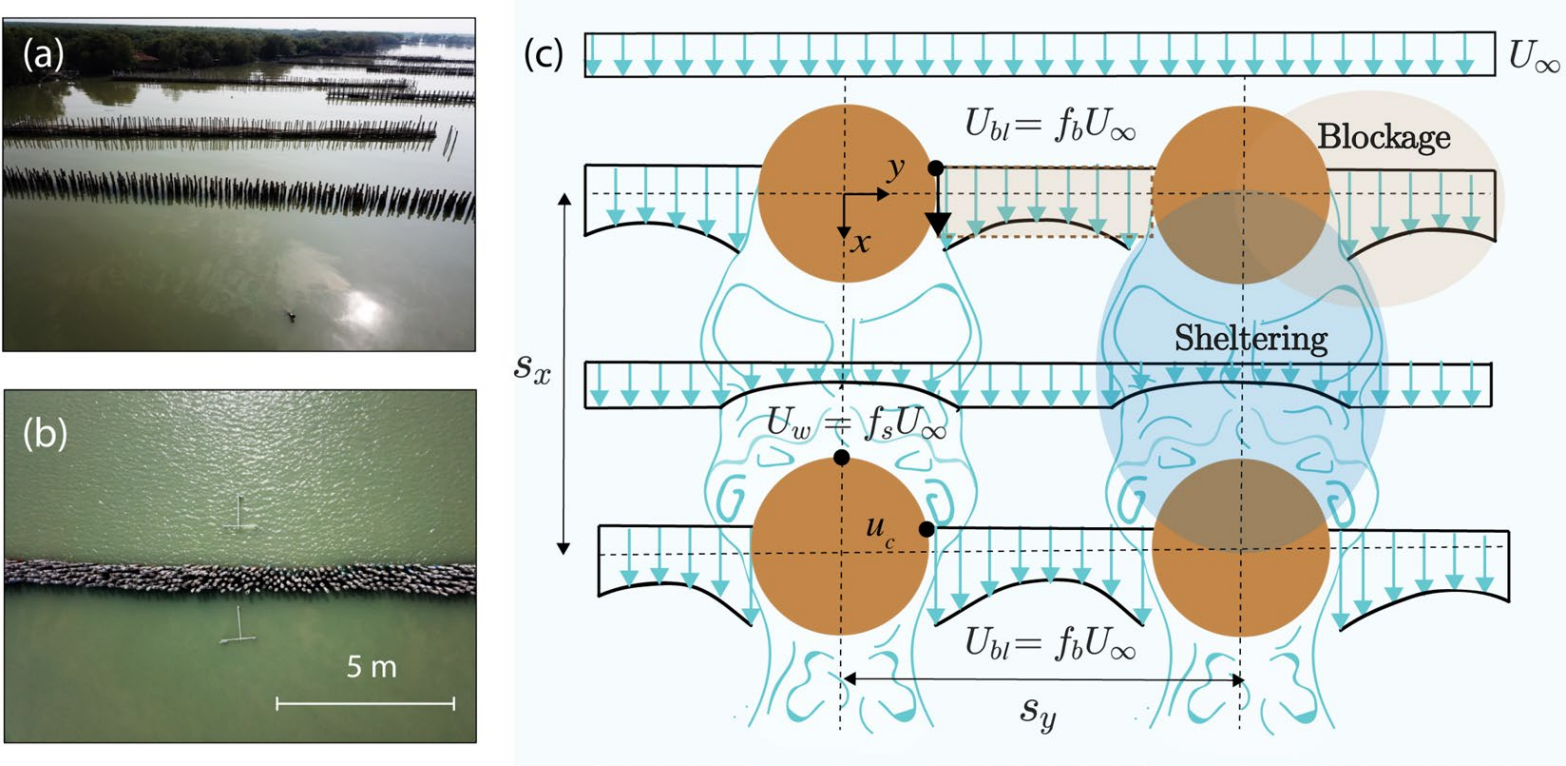
(**a**) Pictures of bamboo structures built by the Indonesian Ministry of Marine Affairs and Fisheries in Demak, Indonesia. The structures are formed by arrays of bamboo poles with a diameter of $d \approx$ 0.15 m, distributed over a width of approximately 1.5 m in the streamwise direction. Their volumetric porosity ranges between $n \approx$ 0.5 and 0.9. (**b**) Top view of one of the structures. Both drone pictures are courtesy of S.A.J. Tas. (**c**) Sketch representing the effects of blockage and sheltering on the local flow velocity (blue arrows) at the scale of the bamboo poles of a structure (solid brown circles), inspired by Etminan et al.^11^ and Zdravkovich^12^. Incoming flow velocities $U_{\infty }$ accelerate to $U_{bl}$ between the cylinders, an effect known as blockage. Behind the first row of cylinders, velocities reduce to $U_w$ due to sheltering effects. The relative magnitude of these effects depends on the streamwise and lateral or spanwise spacing of the cylinders ($s_x$ and $s_y$, respectively).

Mangrove forests effectively function as extensive wood fences that protect coastal communities from storms^1,2^ by attenuating waves and currents, and by preventing erosion^3^. Regardless of their economic and societal value, $30\%$ of the mangrove forests have disappeared around the world over the last 50 years^4^. Mangrove deforestation can increase the exposure of the remaining forest to wave action, causing coastline retreat, and hindering the natural recovery of the forest^5^. Bamboo and brushwood structures have consequently been built to counteract erosion at degraded mangrove sites in South East Asia and South America^3,5–9^. Some of the configurations constructed in a pilot project in Indonesia, consisting of groups of cylindrical bamboo poles driven into the soil, are presented in Fig. 1. The width of the structures varies between 0.7 and 1.5 m in the flow direction, and their volumetric porosity ranges between $n \approx$ 0.5 and 0.9, where *n* is defined as the ratio of the fluid volume to the total volume. Since waves lose energy as they pass through the structures, the calmer hydrodynamic conditions behind the poles enhance sediment deposition, and favour mangrove expansion^5^. Although the structures are designed for wave attenuation, they can also affect local currents, which in turns influences sediment transport and mangrove habitat creation. However, this aspect has received less attention in existing designs^10^. Predicting the impact of the bamboo structures on spatial flow patterns requires quantifying the resistance forces exerted by the structures in currents. The aim of this study is thus to develop a design tool t o calculate this resistance, which could be implemented in large-scale flow models to optimize the performance of future designs.

When a current encounters a structure, flow separation causes form drag forces on the individual poles, and the associated energy dissipation. The drag forces depend on the local flow velocities inside the structure, and on an empirical drag coefficient, $c_D$. The drag coefficient depends on object geometry (surface roughness, cross-sectional shape, height compared to the water depth), and on the flow regime, usually classified as viscous or turbulent^13^. For a circular cylinder in turbulent flow, $c_D$ takes a value of approximately 1^13^. The local flow velocities will vary depending on the arrangement of the poles. On one hand, the presence of the poles reduces the available cross-sectional fluid area (“blocking the flow”), which increases the velocities between the elements due to mass conservation. This effect is referred to as blockage^11,14–16^. On the other hand, downstream elements may be sheltered by upstream wakes, which reduces the velocities acting on them^12,17–20^. This second effect is referred to as sheltering. The relative importance of these two processes will depend on the flow conditions, and on the size and distance between the poles, as illustrated in Fig. 1c.

**Figure 2:**
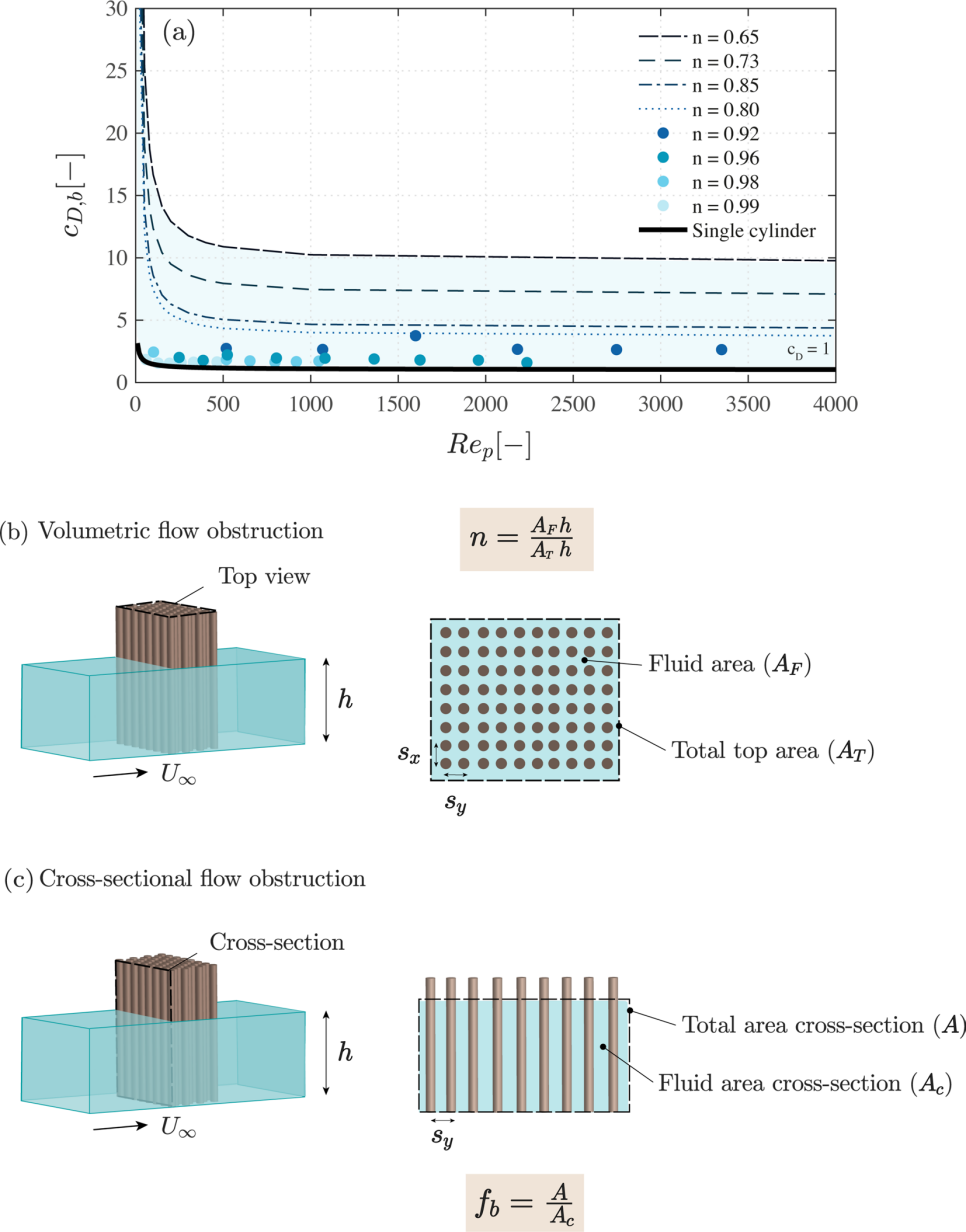
(**a**) Bulk drag coefficient values ($c_{D,b}$) from the literature (including blockage and sheltering effects) as a function of the Reynolds number based on the cylinder diameter and pore velocities ($Re_p$). The shaded blue area shows the region of variation of $c_{D,b}$. The data was collected for arrays of emergent and smooth circular cylinders in a current. The arrays fully covered the cross-section of the flume. The exact values of porosity, cylinder diameter, flow velocities, and bulk drag coefficient are provided in Table 1. The measurements with volumetric porosities between *n* = 0.92 and 0.99 were obtained from Tinoco and Cowen^22^, and the fit lines for *n* = 0.65–0.80 from Tanino and Nepf^21^. (**b**) Definition of the volumetric porosity *n*, given as the ratio between the fluid volume, $V_F= h A_F$ over the total volume $V = h A$. (**c**) Definition of the blockage factor $f_b$, given as the ratio between the total cross-sectional area *A* of the array and the constrained flow section, $A_c$.

Blockage and sheltering effects are often combined into a single fitting parameter, the bulk drag coefficient, $c_{D,b}$. Several authors have referred the drag forces measured inside cylinder arrays to bulk channel velocities $U_b$, and used $c_{D,b}$ as a fitting factor. The subscript *b* indicates that $c_D$ is referred to the bulk velocities, estimated as $U_b=Q/(w h)$, where *Q* is the total flow discharge, *w* is the channel width, and *h* the water depth. $c_{D,b}$ values from Tanino and Nepf^21^ and Tinoco and Cowen^22^ are shown in Fig. 2a as a function of $Re_p = U_pd/\nu$, where $Re_p$ is the the Reynolds number based on the average pore velocities, $U_p$, and the cylinder diameter *d*. The pore velocity is defined as the velocity averaged over the pore space, which can be estimated as $U_p = U_b/n$, where $U_b$ is the bulk velocity and *n* is the volumetric porosity^21^.

Application of the coefficients of Fig. 2a into designs is not straightforward, due to the variability of $c_{D,b}$ for a fixed value of $Re_p$. For instance, $c_{D,b}$ varies between 1 and 10 for $Re_p = 1000$ in Fig. 2a, and referring the drag forces to the bulk velocities does not enable distinguishing how blockage and sheltering effects led to different drag values for the same $Re_p$. A number of authors have proposed relating the drag forces to the pore velocities $U_p$, and considering these as representative of the flow conditions inside the structures^21–23^. The concept of pore velocity is based on mass conservation over the fluid volume, and it is illustrated in Fig. 2b. The drag coefficient based on $U_p$ reduces the variability of the fitted drag to $c_{D,p} = 1-4$ for the conditions of Fig. 2a with $Re_p = 1000$, but it still leaves too much uncertainty in the choice of the coefficient. This led to the research work of Etminan et al.^11^, who suggested that the variability in drag measurements could be due to the local velocities between cylinders exceeding $U_p$, and causing consequently higher bulk drag coefficients. Their modelling work showed that the drag forces were better represented by the constrained velocities, calculated from mass conservation at a cross-section of the flow, as shown in Fig. 2c. Etminan et al.^11^ modelled conditions that corresponded with natural vegetation, with a volumetric porosity of *n* = 0.78–0.98, where sheltering effects were very small. Incorporating sheltering effects in drag predictions may be necessary for the bamboo structures, which are relatively less porous with $n =$ 0.5–0.9.

A number of (semi)empirical approaches have been derived to integrate sheltering effects in the predictions of the drag forces. Blevins^24^ developed an expression for the velocity deficit on a downstream cylinder based on wake similarity laws, for two cylinders in cross-flow. Higher turbulence levels are expected inside an array with more elements^25^, and this factor has been observed to influence the rates of velocity decay behind cylinders^26^. Eames et al.^26^ developed a model for the velocity deficit behind a cylinder that also included the effect of ambient turbulence, but the application of this model for the bamboo structures would require a separate module to calculate turbulent production between the cylinders. Meftah and Mossa^27^ developed a model for the flow velocity reduction inside sparse cylinder arrays, relating the velocity deficit behind the cylinders with the geometrical properties of the array, and with an empirical turbulent mixing length scale *l*. However, due to the lower porosity of the bamboo structures, and the smaller relative distance between their poles, blockage is likely to influence turbulence production, which sets the bamboo structures outside the range of calibrated data and the assumptions of the previous models. Quantifying sheltering effects for the bamboo structures thus requires adapting the existing approaches.

We consequently present a physics-based model to predict the drag forces acting on emergent cylinder arrays exposed to currents, which provides a direct relationship between cylinder arrangement and $c_{D,b}$. The velocities inside the arrays are estimated using a blockage factor, based on mass conservation, and a sheltering factor, based on the wake flow model developed by Eames et al.^26^. Since the model of Eames et al.^26^ requires knowledge of the ambient turbulence intensity, we expand the turbulence model of Nepf^25^, including a turbulence production term by flow expansion, as done by Mossa et al.^28^, and the effects of blockage and sheltering in the wake production term. This model focuses on the local physical processes inside the structures, and it computes the bulk hydrodynamic forcing using the incoming flow velocity and flow depth as input parameters. In order to calculate the effects of the structures on the surrounding flow field (such as backwater effects or changes in the flow direction in coastal regions), the equations of the model could be built in standard free surface flow models that solve for those processes. The development of the bulk drag model is presented in the next section. Following its derivation, the model is tested against force measurements from random cylinder arrays by Tanino and Nepf^21^ and Tinoco and Cowen^22^, and from regular cylinder arrays by Jansen^29^. The experiments of Jansen^29^ are described in “Methods” section. The model behaviour is also explored for different cylinder configurations. Finally, the model sensitivity to different input parameters is investigated, and the model is applied to optimize future structure designs.

## Model development

The analytical model consists of (1) an adapted drag formulation for closely-packed cylinder arrays, including blockage and sheltering, and (2) a turbulent kinetic energy balance, necessary to quantify sheltering. The turbulence model builds on the formulation suggested by Nepf^25^ for vegetation canopies, and incorporates a turbulence production term by flow expansion, and an extended drag formulation in the wake production term. The steps to derive the equations are presented below.

### Drag model

The drag forces experienced by an array of cylinders, per unit mass, can be calculated as:

1 $$\begin{aligned} \ F_{d} = \frac{1}{2}c_D a |U|U \ \end{aligned}$$

where $c_D$ is the drag coefficient of a single cylinder, which can be estimated using the empirical expression of White^30^, given by:

2 $$\begin{aligned} \ c_D = 1 + 10Re^{-2/3} \ \end{aligned}$$

where *Re* is the Reynolds number based on the cylinder diameter and the depth-averaged local flow velocity *U*. *a* is the projected plant area per unit volume, defined by Nepf^25^ as:

3 $$\begin{aligned} \ a = \frac{d h}{h s^2} = \frac{d}{s^2}\ \end{aligned}$$

with *d* being the cylinder diameter, *s* the spacing between cylinders, and *h* the water depth.

The main unknown in Eq. (1) is the local flow velocity *U*. If a cylinder array is sufficiently sparse, the local flow velocity could be assumed equal to the depth-averaged incoming flow velocity, $U_{\infty }$, either measured or calculated with a free surface flow model. For denser configurations, the velocity will change as the flow propagates through the array due to (1) flow acceleration between the elements (blockage), and (2) flow deceleration due to the sheltering effects of upstream rows of cylinders. Both effects are illustrated in Fig. 1c. The changes in flow velocity are included by multiplying $U_{\infty }$ by a blockage factor, $f_b$, and a sheltering factor, $f_s$:

4 $$\begin{aligned} \ U = f_b f_s U_{\infty }\ \end{aligned}$$

Inserting both factors in the expression for the drag force results in Eq. (5):

5 $$\begin{aligned} \ F_{d} = \frac{1}{2}c_D a |U|U = \frac{1}{2}c_D a f_b^2 f_s^2 |U_{\infty }|U_{\infty } = \frac{1}{2} c_{D,b} a |U_{\infty }|U_{\infty } \ \end{aligned}$$

where the changes in velocity have been incorporated in the bulk drag coefficient, $c_{D,b} = c_D f_b^2 f_s^2$. This expression provides a direct relationship between the drag coefficient of a single cylinder, $c_D$, and bulk drag coefficients $c_{D,b}$ measured for cylinder arrays in laboratory experiments. Predicting the drag force thus depends on determining the values of $f_b$ and $f_s$.

The blockage factor $f_b$ can be estimated based on mass conservation through a row of cylinders^11^, considering that the velocity will increase as the same flow discharge travels through the smaller section between the elements:

6 $$\begin{aligned} \ U_{\infty } A = U_c A_c = f_b U_{\infty } A_c \ \end{aligned}$$

where the total frontal area is $A = h s_y$, and $s_y$ is the distance between cylinders perpendicular to the flow, center-to-center (see Fig. 1). Subtracting the frontal area of the cylinders from the total area gives the available flow area, $A_c$:

7 $$\begin{aligned} \ A_c = h s_y - h D = h (s_y-d) \ \end{aligned}$$

Here we are assuming that the water depth is the same just upstream and in between the cylinders. Solving for $f_b$ in Eq. (6) results in Eq. (8), see also Etminan et al.^11^:

8 $$\begin{aligned} \ f_b = \frac{h s_y}{ h (s_y-d)} = \frac{1}{1-d/s_y} \ \end{aligned}$$

The sheltering factor $f_s$ can be estimated from the wake flow model by Eames et al.^26^, which predicts the velocity deficit behind a cylinder as a function of the distance downstream of the cylinder, $s_x$, the cylinder diameter, the local turbulent intensity $I_t$, and the drag coefficient:

9 $$\begin{aligned} \ \frac{U_{\infty }-U_{w}}{U_{\infty }} = \frac{c_D d}{2\sqrt{2 \pi } I_t s_x}\ \end{aligned}$$

where $U_{w}$ is the velocity in the cylinder wake, $U_{\infty }$ is the incoming flow velocity, and $I_t$ is the meant turbulent intensity, defined as $I_t = \sqrt{k}/U_{\infty }$^21,25^. *k* represents the turbulent kinetic energy per unit mass, with $k = 1/2(\overline{u'^{2}} + \overline{v'^{2}} + \overline{w'^{2}})$, where $u'$, $v'$, and $w'$ are the instantaneous velocity fluctuations in the streamwise, lateral, and vertical direction respectively, and where the overbar denotes time averaging. The turbulent velocity fluctuations are defined as the difference between the instantaneous velocities and their mean value over a measurement period. Here we consider the depth-averaged value of the turbulent intensity, in view of the uniformity of the turbulent properties over the vertical observed inside emergent arrays^25^.

Equation (9) was developed assuming turbulent flow. Viscous effects decrease the velocity deficit^26^, with the reduction factor being given by:

10 $$\begin{aligned} \ f_{Re} = \sqrt{\frac{Re}{Re_{t}}} \ \end{aligned}$$

where $Re_{t}$ is the lowest Reynolds number corresponding to fully turbulent wake flow. Laminar effects are included in the wake flow model by multiplying the velocity deficit of Eq. (9) by the reduction factor $f_{Re}$ for $Re < Re_t$, where the the turbulent Reynolds number is assumed equal to $Re_t = 1,000$. This value is based on the observation that although a wake starts becoming turbulent at $Re_{t} \sim 200$, drag coefficient measurements usually become constant at Reynolds numbers beyond $Re_{t} \sim 1000$, e.g. as shown in Figure 2.7 of Sumer and Fredsoe^13^. The influence of varying $Re_{t}$ on the model results is investigated in “Results and discussion” section.

Defining the sheltering factor as $f_s = \frac{U_{w}}{U_{\infty }}$, and including $f_{Re}$ and the bulk drag coefficient in the definition of the velocity deficit results in Eq. (11):

11 $$\begin{aligned} \ f_s = \frac{U_{w}}{U_{\infty }} = 1-f_{Re}\frac{c_{D,b} d}{2\sqrt{2 \pi } I_t s_x} = 1-f_{Re}\frac{c_{D,b} d}{2\sqrt{2 \pi } (\sqrt{k}/U_{\infty }) s_x}\ \end{aligned}$$

Equation (9) also assumes that the downstream cylinder is placed inside the ballistic spreading region of the wake. The ballistic regime occurs for a downstream distance $s_x < L/It$, where *L* is the integral length-scale of turbulence, and it is characterized by a rapidly decaying velocity deficit, and by a linear increase of the wake width with downstream distance. Inside the cylinder arrays, the length scale development is limited by the downstream spacing, resulting in $L < s_x$. Considering that turbulent intensity measurements of Jansen^29^ varied between $I_t$ = 0 and 0.8 inside cylinder arrays with *n* = 0.64–0.9, this would result in $L < s_x/It$. This is a reasonable general assumption for the bamboo structures, since their porosity varies in a similar range. If the poles were sparsely placed, there would be a transition from ballistic to diffusive spreading of the wake. Eames et al.^26^ also developed expressions for turbulent flow under the diffusive regime, which could be used in place of Eq. (9).

In the opposite case of very high pole densities, there may be a point where the elements are so closely-packed that vortex shedding is inhibited by the presence of the neighboring cylinders. Considering an analogy with a cylinder placed close to a solid boundary, vortex shedding would not take place for spanwise spacings smaller than $s_y/d < 1.3$^13^, causing a decrease of the drag coefficient that would not be reproduced by the expression of White^30^. The application of the present model is thus restricted to $s_y/d > 1.3$.

### Balance of turbulent kinetic energy

Application of Eq. (11) requires predicting the turbulent kinetic energy. This is calculated by expanding the model developed by Nepf^25^, based on a balance between turbulence production and dissipation:

12 $$\begin{aligned} \ P_w \sim \epsilon \ \end{aligned}$$

where $P_w$ is the turbulent production rate and $\epsilon$ is the dissipation rate. For a dense cylinder array, *k* is produced by (1) generation in the wakes of the cylinders^25^, and (2) shear production by the jets formed between the elements^28^. The total turbulence production term, $P_w$, consequently has two parts:

13 $$\begin{aligned} \ P_w = P_{w1}+P_{w2} \ \end{aligned}$$

We assume that for dense cylinder arrays these two terms are much higher than turbulence production by shear at the bed, based on observations by Nepf^25^ for sparse arrays. This assumption is further tested in “Results and discussion” section.

The first term in Eq. (13), $P_{w1}$, represents turbulence production at the wakes, and can be estimated as the work done by the drag force times the local flow velocity:

14 $$\begin{aligned} \ P_{w1} = \frac{1}{2}c_D a |U|U^2 = \frac{1}{2}c_D a f_b^3 f_s^3 |U_{\infty }|U_{\infty }^2 \ \end{aligned}$$

The second term, $P_{w2}$, represents turbulence generation due to flow expansion^28^, and can be estimated from the Reynolds shear stresses:

15 $$\begin{aligned} \ P_{w2} = \overline{ u' v'} \frac{\partial u }{\partial y} \ \end{aligned}$$

where the overbar denotes time averaging. The loss in mean kinetic energy $E_c$ due to flow expansion is equal to:

16 $$\begin{aligned} \ \Delta E_c = \frac{1}{2} U_{\infty }^2 \left( \left( \frac{A}{A_c}\right) ^{2}-1 \right) = \frac{1}{2} \left( f_b^{2}-1 \right) U_{\infty }^2\ \end{aligned}$$

where the energy loss due to flow expansion, $\Delta E_c$, is modelled using the Carnot losses. Assuming that the mean kinetic energy is transformed into turbulent kinetic energy $E_t$, and assuming isotropic turbulence, gives Eq. (17):

17 $$\begin{aligned} \ \frac{1}{2} \left( f_b^{2}-1 \right) U_{\infty }^2 = \frac{3}{2}\overline{ u' u'}\ \end{aligned}$$

Equation (17) enables expressing the normal Reynolds stress as a function of the incoming flow velocities and the blockage factor:

18 $$\begin{aligned} \ \overline{ u' u'} = \frac{1}{3} \left( f_b^{2}-1 \right) U_{\infty }^2 \ \end{aligned}$$

The Reynolds shear stress is estimated as $\overline{ u' v'} = R\overline{ u' u'}$, where the correlation factor *R* was given a constant value of 0.4 based on observations of Nezu and Nakagawa^31^. This value was derived for open channel flow conditions and is assumed acceptable as a first approximation, but it could vary inside a cylinder array. This is explored further in “Results and discussion” section.

The velocity gradient is estimated from the velocity difference between the side of the cylinders (dominated by blockage) and the wake of the cylinders (dominated by sheltering) resulting in Eq. (19):

19 $$\begin{aligned} \ \frac{\partial u }{\partial y} \approx \frac{U_{\infty }(f_b-f_s)}{\frac{1}{2} s_y} \ \end{aligned}$$

Substitution into Eq. (15) gives Eq. (20):

20 $$\begin{aligned} \ P_{w2} = \frac{2}{3} R (f_b-f_s)(f_b^{2}-1)\frac{U_{\infty }^3}{s_y} \ \end{aligned}$$

The dissipation term, $\epsilon$, is estimated as:

21 $$\begin{aligned} \ \epsilon \sim k^{3/2} l^{-1} \ \end{aligned}$$

The characteristic turbulent length scale *l* is limited by the surface-to-surface separation between the elements in the flow direction, $l = min(|s_x-d|, d)$. This differs from the expression developed by Nepf^25^, who used the diameter as representative of the size of the eddies. We assume that in closely-packed cylinder arrays the spacing between cylinders may be smaller than the diameter, $|s_x-d| < d$, which would limit turbulence development. The maximum value of *l* is set equal to the cylinder diameter. Here we also assume that for the dense cylinder arrangements, the spacing between cylinders is considerably smaller than the water depth, hence turbulence generated by bed friction is negligible.

Balancing the production and dissipation of turbulent kinetic energy results in Eq. (22):

22 $$\begin{aligned} \ \frac{k^{3/2}}{l} \sim |U_{\infty }|U_{\infty }^2\left( c_D a f_b^3 f_s^3 + \frac{ 4R}{3s_y}(f_b^{2}-1)(f_b-f_s)\right) \ \end{aligned}$$

Taking the cubic root at both sides and introducing the scale factor $\alpha _1$ gives Eq. (23):

23 $$\begin{aligned} \ \frac{\sqrt{k}}{U_{\infty }} = \alpha _1\left( c_D f_b^3 f_s^3 a l + \frac{4}{3}R(f_b^{2}-1)(f_b-f_s)\frac{ l}{s_y}\right) ^{1/3} \ \end{aligned}$$

Where $\alpha _1$ is a coefficient of ${\mathcal {O}}(1)$, which is given a default value of $\alpha _1 = 1$. The sensitivity of the model to different $\alpha _1$ and *R* values is explored in “Results and discussion” section.

*k* can be calculated by solving Eq. (23) iteratively, using the incoming upstream velocity $U_{\infty }$ and the geometric characteristics of the structure, $s_y, s_x, d$ and *a*, as an input. This enables determining the sheltering factor, $f_s = U_{w}/U_{\infty }$ from Eq. (11). The blockage factor $f_b=(1-d/s_y)^{-1}$ can also be calculated from the geometric properties of each configuration. Both coefficients can be then combined to predict the bulk drag coefficient, with $c_D,_{b} =c_D(f_s)^2(f_b)^2$. Deriving $c_D,_{b}$ with the present approach relies on the assumption that the changes in water depth through the structure are small. This is a reasonable assumption given the short length of the bamboo structures in the streamwise direction, which varies between 0.7 and 1.5 m (see Fig. 1b). Longer structures that experience non-negligible changes in flow depth and velocity should be discretized, and the bulk drag coefficient should be calculated separately for the different sections. The model assumptions are discussed further in the following section.

## Results and discussion

In this section we firstly present the model validation, and investigate how turbulence production and sheltering vary under different configurations. We then explore the model sensitivity to several input parameters, and finally apply the model to investigate structure design optimization.

### Model validation

The performance of the model is tested against drag measurements for regular, staggered and random emergent cylinder arrangements from the literature. A summary of the conditions tested in the different studies is shown in Table 1. The regular configurations, also denoted as in-line arrangements, consist of rows of cylinders where the downstream elements are always in one line in the streamwise direction (see configurations 1–6 tested by Jansen^29^ in Fig. 7 b of “Methods” section). In the staggered arrangements, for every row the downstream elements are shifted laterally so that they are located at the center line of upstream elements, as also shown in configuration 7 of Fig. 7b. The random arrangements were obtained by distributing the cylinders using a random number generator, see Tanino and Nepf^21^.

**Table 1:** Validation data for emergent cylinder arrays.

Source	Arrangement	$s_x/d$ [–]	$s_y/d$ [–]	*d* [m]	*n* [–]	$U_{\infty }$ [m/s]	$c_{D,b}$ [–]
Tinoco and Cowen^22^	Random	2.39	2.39	0.0025	0.92	0.16	2.85
		2.89	2.89	0.013	0.96	0.22	1.90
		3.67	3.67	0.006	0.98	0.22	1.83
		4.83	4.83	0.003	0.99	0.20	1.64
Tanino and Nepf^21^	Random	2.29	2.29	0.006	0.80	0.05	2.63
		1.98	1.98	0.006	0.85	0.06	2.71
		1.71	1.71	0.006	0.73	0.03	3.61
		1.50	1.50	0.006	0.65	0.03	3.93
Jansen^29^	Regular	$\infty$	1.50	0.040	0.33	0.40	8.98
		3.00	1.50	0.040	0.79	0.40	6.19
		1.50	1.50	0.040	0.64	0.40	4.44
		$\infty$	3.00	0.040	0.66	0.40	2.67
		3.00	3.00	0.040	0.90	0.40	1.59
	Staggered	3.00	3.00	0.040	0.82	0.40	1.93

**Figure 3:**
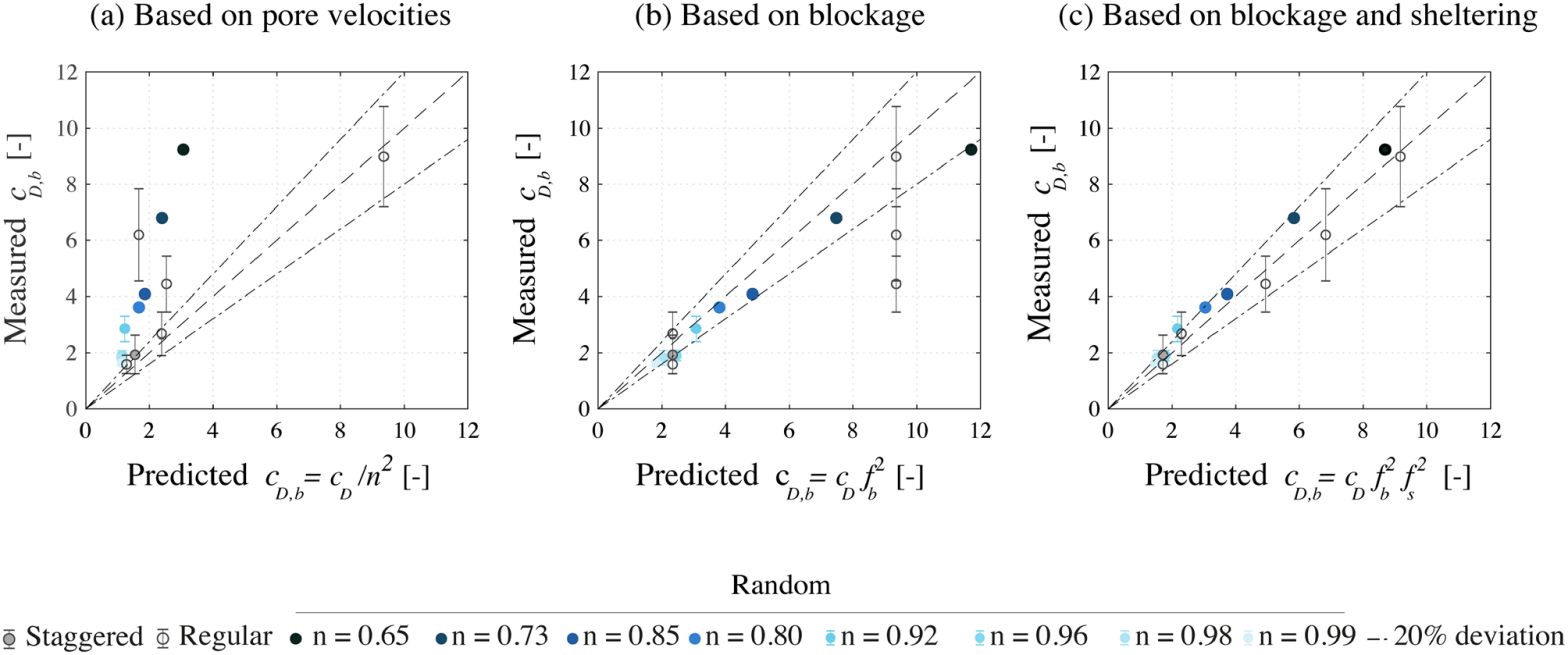
Predictions of the analytical model versus $c_{D,b}$ measurements for random cylinder arrays with varying porosities, by Tinoco and Cowen^22^ and Tanino and Nepf^21^, and versus measurements for regular and staggered arrangements by Jansen^29^. Plot (**a**) shows the bulk drag coefficient calculated from the pore velocities (based on mass conservation over the fluid volume). Plot (**b**) shows the drag values derived from blockage factor (based on mass conservation over a cross-section). Plot (**c**) shows the results of the present model, which includes both blockage and sheltering effects. The vertical bars show the estimated measurement error.

In Fig. 3 we compare the model predictions for the cases of Table 1 with two other approaches used in the literature to define the bulk drag coefficient. Figure 3a shows the bulk drag coefficient calculated from the pore velocities (based on mass conservation over the fluid volume). Figure 3b shows the drag values derived from blockage factor (based on mass conservation over a cross-section, from Eq. 7). Figure 3c shows the results of the present model, which includes both blockage and sheltering effects.

Using the pore velocities to estimate the bulk drag results in a general under-estimation of the drag coefficients (Fig. 3a). The blockage factor provides better estimates of the bulk drag for random arrays, but it cannot reproduce the sheltering effects observed at regular arrangements with different streamwise separations (Fig. 3b). The present model, including both sheltering and blockage, successfully reproduces the bulk drag for regular configurations, and it also provides a slight improvement for the random arrangements (Fig. 3c). The model displays a general tendency to overestimate the bulk drag of staggered and random configurations, which could be due to changes in the flow direction through such configurations.

Random and staggered arrangements have been associated to similar bulk drag coefficients in the literature^32^, which were higher than for regular configurations^33–36^. Schoneboom et al.^36^ attributed the larger drag for staggered arrays to the more tortuous water flow through them. The present model assumes that the flow propagates only in the streamwise direction, and that it does not experience changes in direction. This assumption still yielded good results with the validation, especially for the densest configurations. This is expected because as the element density increases most of the total volume is occupied by cylinders. Less room for varying the spatial arrangement results in similar drag forces for regular and random arrays.

Although the model does not include changes in water level through the structures, it could still reproduce the measurements of Tanino and Nepf^21^ and Tinoco and Cowen^22^, conducted with array lengths of 0.99 m and 2.84 m, respectively. This assumption may not hold for longer cylinder arrays over a fixed horizontal bed. Under those conditions the water depth could experience significant changes through the structure, which should be taken into account in bulk drag predictions. However, since the bamboo structures have a short length in the streamwise direction, such cases are beyond the scope of the present work.

### Influence of spacing on hydrodynamic parameters

**Figure 4:**
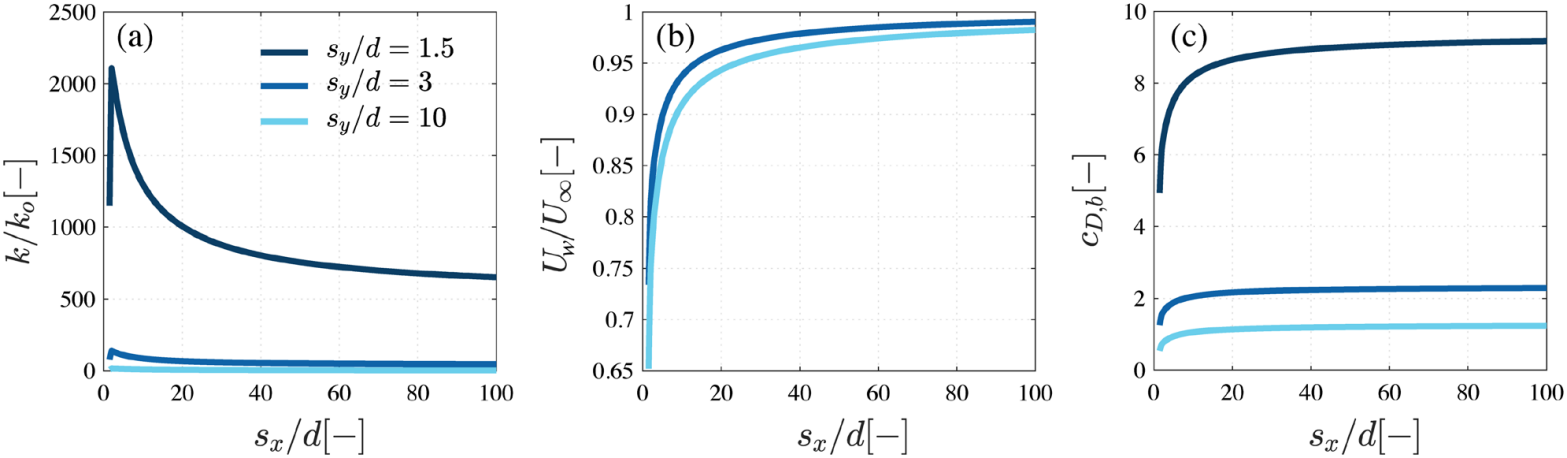
Model results for (**a**) the turbulent kinetic energy *k* compared to the turbulent production of a bare smooth bed, $k_o$, for (**b**) the sheltering factor, $U_w/U_\infty$, and for (**c**) the bulk drag coefficient $c_{D,b}$ as a function of the streamwise spacing $s_x$ and spanwise spacing $s_y$ between cylinders compared to the cylinder diameter, *d*. The lines for $s_y/d = 1.5$ and $s_y/d = 3$ are on top of each other in plot (**b**). The figure shows that smaller spanwise spacings $s_y$ result in higher turbulence production and faster wake recovery. The smaller sheltering effects combined with larger flow acceleration result in higher bulk drag coefficients for low $s_y$.

Once validated, the model is applied to investigate the influence of the distance between elements on turbulence production and sheltering, and to evaluate how the previous effects translate into different $c_{D,b}$ values. Figure 4 shows the turbulent kinetic energy, sheltering factor, and bulk drag coefficient calculated for three values of spanwise spacing, $s_y/d = 1.5, 3$ and 10, for streamwise separations between $s_x/d = [1,100]$.

The turbulent kinetic energy, shown in Fig. 4a, is expressed as a ratio to the turbulent kinetic energy produced by bottom friction, $k_o$. Turbulence generation at the bed is based on the friction velocity with $k_o = c_{f,b} U_{\infty }^2$, where $c_{f,b} = 0.001$ corresponding to a smooth bottom. Overall, the levels of turbulence inside cylinder arrays are considerably higher than for a bare bed. The turbulent kinetic energy increases with smaller spanwise spacing $s_y/d$, since blockage increases the drag forces, their work, and the shear production term. The largest spanwise spacing, $s_y/d = 10$, produces relatively lower values of *k*, but these are still between $k =[4-20]k_o$. The turbulence levels also vary as a function of the streamwise spacing, decreasing their values for the lowest $s_x/d$, since sheltering effects cause a strong reduction of the turbulence production terms. Higher streamwise separations reduce sheltering effects, and increase turbulence production up to a relative maximum around $s_x/d \sim 2$. Beyond the maximum, the larger streamwise separations are associated to a lower number of cylinders per unit volume, a smaller projected area *a*, and less production of turbulent kinetic energy per unit mass.

These trends are also visible in the sheltering factor, shown in Fig. 4b, as the velocity deficit over a cylinder is inversely proportional to the level of ambient turbulence. The velocity deficit is consequently smaller for low $s_y/d$ values. Since the velocity reduction is also inversely proportional to $s_x/d$, sheltering effects are less pronounced for higher $s_x/d$ values. This results in the bulk drag coefficients, shown in Fig. 4c, being governed by the blockage factor for $s_x/d > 15$, and by both sheltering and blockage for lower $s_x/d$ values.

### Sensitivity analysis

**Figure 5:**
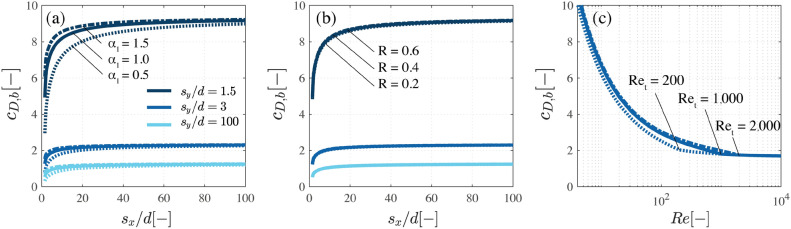
Sensitivity of the modelled bulk drag coefficient $c_{D,b}$ to varying values of (**a**) the scale factor $\alpha _1$ and (**b**) the correlation factor *R*, as a function of the streamwise spacing $s_x$ and spanwise spacing $s_y$ between cylinders compared to the cylinder diameter, *d*. Plot (**c**) shows the influence of the limit for turbulent flow, $Re_t$, on the bulk drag predictions for $s_y/d = 1.5$ as a function of the Reynolds number *Re*.

The present model depends on the values that are assumed for the parameters $\alpha _1$, *R* and $Re_t$. The model sensitivity to changes around their default values is explored in Fig. 5.

The scale factor $\alpha _1$ is varied between 0.5 and 1.5 in Fig. 5a. The lower limit of $\alpha _1 = 0.5$ is associated to a relatively low turbulence production, which in turn increases the velocity deficit on downstream elements. This results in considerable sheltering effects up to $s_x/d \sim 40$. $\alpha _1 = 1$ increases turbulence production and reduces the velocity deficit, causing appreciable sheltering effects up to $s_x/d \sim 20$. The higher value of $\alpha _1 = 1.5$ provides comparable results to $\alpha _1 = 1$. A more precise assessment of $\alpha _1$ would require measurements of turbulence production and dissipation inside different cylinder configurations. Since laboratory measurements presented in the literature show that sheltering effects can be evident at a downstream distance of $s_x/d = 15$^18^, it is concluded that $\alpha _1 = 1$ provides reasonable predictions of the sheltering effect. As shown in Fig. 5b, the model results display low sensitivity to variations of the factor *R*, since the shear production term $P_{w2}$ has a relatively lower weight on the total turbulence production in comparison with the wake production term $P_{w1}$. The influence of $Re_t$ on the bulk drag predictions is illustrated in Fig. 5c. Lower values of $Re_t$ result in stronger sheltering effects and smaller bulk drag coefficients. The largest difference between the three tested values was observed for $Re = 200$, where $c_{D,b} = 2, 2.5,$ and 2.6 for $Re_t = 200, 1000,$ and 2000, respectively. An accurate evaluation of this threshold would require force and velocity measurements inside cylinder arrays with Reynolds numbers varying in the previous $Re_t$ range. Considering the large diameter of the bamboo poles, the Reynolds numbers in the field are most likely to be of the order of $Re \sim 10,000$. This implies that the $Re_t$ threshold will not affect significantly the drag force predictions for the structures.

## Drag maximization

**Figure 6:**
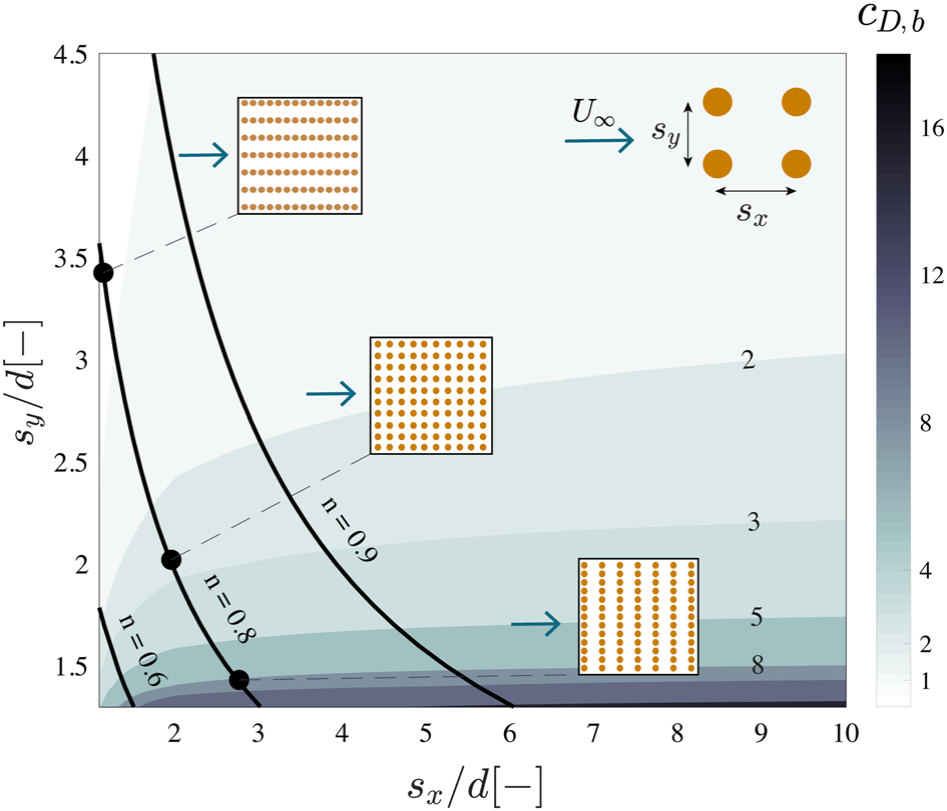
Predicted bulk drag coefficient as a function of the streamwise spacing $s_x$ and spanwise spacing $s_y$ between cylinders compared to the cylinder diameter, *d*. The diagram shows lines of constant volumetric porosity *n*. Three examples of regularly spaced configurations with constant porosity of $n=0.8$ but varying streamwise/spanwise spacing are given. Flow direction relative to the arrays is indicated by a blue arrow (from left to right). The diagram shows that given a constant porosity, higher drag values can be obtained for smaller spanwise spacings $s_y$ and longer streamwise spacings $s_x$.

The choice of pole configuration, in terms of element spacing $s_x/d$ and $s_y/d$, is thus essential to assess the bulk drag and the resistance provided by a structure. This is conceptualized in Fig. 6.

Figure 6 illustrates the computed bulk drag coefficient for different combinations of the dimensionless spacing $s_x/d$ and $s_y/d$. The lowest value of $s_y/d$ is limited to 1.3 since, as previously discussed, below that value the expression of White^30^ may not be valid. We also include solid black lines showing configurations with the same volumetric porosity. Figure 6 shows that a structure with a porosity of $80\%$ can have an average bulk drag coefficient between $c_{D,b}$ = 1 and 10 depending on the element placement. The highest bulk drag coefficients are associated to rows of cylinders with a small spanwise spacing $s_y/d$, which enhances blockage, and a large streamwise spacing $s_x/d$, so that downstream rows experience less velocity reduction. For instance a regular structure with $80\%$ porosity and a spanwise spacing of $s_y/d = 1.4$, would have a streamwise spacing of $s_x/d = 2.8$. This would result in a bulk drag coefficient of $c_{D,b} = 8$. If the same number of elements were placed in a uniform setting, with $s_x/d = s_y/d = 2$, this would led to a much lower bulk drag coefficient of $c_{D,b} = 3$.

Placing the rows in a staggered manner could reduce sheltering effects, but even assuming negligible sheltering, a spanwise spacing of $s_y/d = 2$ would lead to a bulk drag coefficient of $c_{D,b} = 4$ (with $c_{D,b} = c_D f_b^2$). A similar effect could be achieved with a random configuration, but predicting the net effect of the spatial changes in density on the drag would require more detailed knowledge of the cylinder density distribution. In a random arrangement the flow will tend to deflect to areas of low element density, but its trajectory will also depend on the length of the paths. A shorter path where the cylinder are more densely placed could lead to lower resistance than a longer and sparser alternative^37^. However, as previously discussed, for relatively denser structures, uniform and random arrangements should yield comparable forces.

The present drag model may be implemented in large scale hydrodynamic models to evaluate the impact of currents, and the associated forces, on the cylinders. This approach would enable varying the cylinder arrangement, structure length and location, and help identify parameter combinations that optimize future structure designs. Moreover, although the present model was developed for currents, it is applicable for long waves (with $KC > 100$, where *KC* represents the ratio of wave excursion to pole diameter) where non-stationary effects are negligible. For shorter waves (with $KC < 100$), the hydrodynamic forces also depend on additional aspects, such as inertial effects^38^, or turbulence enhancement by waves^39^. The influence of the previous aspects on the bulk drag coefficient is investigated further in Gijón Mancheño et al.^40^.

## Conclusions

In this study a model is developed to determine the bulk drag coefficient of dense arrays of emergent cylinders, accounting for both blockage and sheltering effects. Flow acceleration through the elements (blockage) is modelled based on mass conservation through a cross-section of the array. The velocity reduction by the wakes of upstream elements (sheltering) is modelled based on the wake flow model of Eames et al.^26^, in combination with an equation to predict turbulence production by the cylinders derived by expanding the model of Nepf^25^. Turbulence production is a function of the spacing between the elements both in the spanwise and streamwise directions. Smaller spanwise spacings increase blockage and turbulence production, while smaller streamwise spacings have the opposite effect; they result in more velocity reduction on downstream cylinders, and less turbulence generation. The differences in turbulent kinetic energy also affect wake recovery, and the velocity deficit of different configurations. Higher levels of ambient turbulence result in smaller velocity deficit behind the cylinders, and less sheltering of downstream elements. When we combine blockage and sheltering effects to predict the bulk drag coefficient, we reproduce measurements from the literature for volumetric porosities between 0.64 and 0.99 within a deviation of 20%. The model also shows that reducing the lateral spacing between elements, and increasing their streamwise separation, increases the bulk drag and the dissipation per element. The application of the present model, and its development for wave flows, could help optimizing future structure designs, minimizing their material costs and erosion problems. The model may thus constitute a practical tool to increase the success of future mangrove restoration schemes.

## Methods

### Validation data

The data that support the findings of this study were directly obtained from the graphs of Tanino and Nepf^21^ and Tinoco and Cowen^22^, and from the dataset collected by Jansen^29^. Jansen^29^ conducted laboratory experiments in the wave and current flume at Delft University of Technology, in order to measure the hydrodynamic forces acting on groups of cylinders with varying geometrical configurations with currents and waves. His report^29^ focuses on the description of flume experiments with waves, and we have thus included a more detailed explanation of the experiments with currents in the present section.

**Figure 7:**
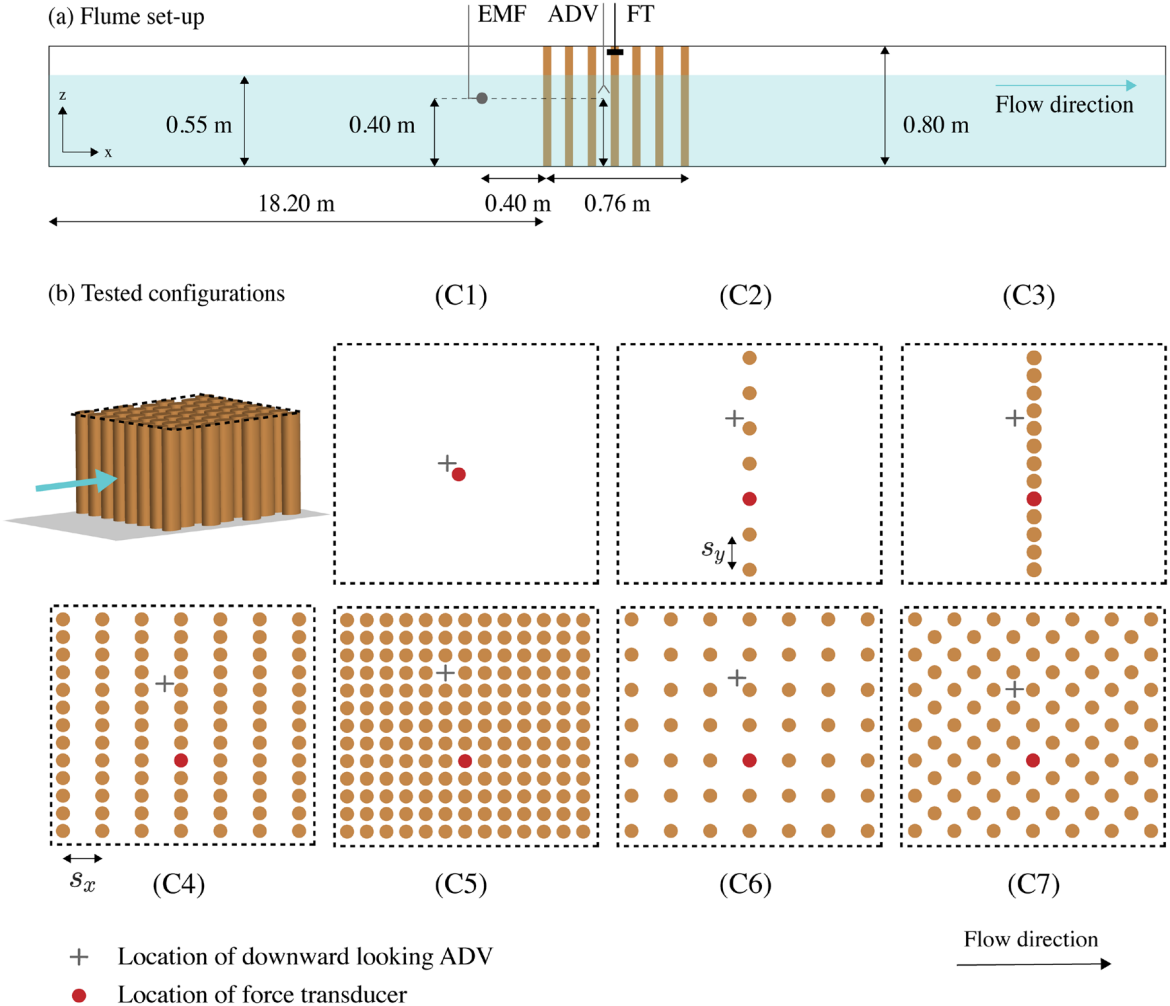
(**a**) Side view of the instrument set-up in the flume, consisting of an electromagnetic flow meter (EMF), a Nortek Vectrino acoustic velocimeter (ADV) and a SCAIME load cell mounted on the upper part of the element (FT). (**b**) Configurations tested in the experiments. An oblique view of the structure is shown at the top left side of the plot, where the flow direction is indicated by a blue arrow. The top view of the structure is marked by a dashed black line, and it is illustrated for each of the tested arrangements: (C1) single cylinder with $d =$ 0.04 m, (C2) single row with spanwise spacing between the elements of $s_y = 3d$, (C3) single row with spanwise spacing between the elements of $s_y = 1.5d$, (C4) multiple rows with $s_y = 1.5d$ and $s_x = 3d$ in uniform arrangement, (C5) multiple rows with $s_y = 1.5d$ and $s_x = 1.5 d$ in uniform arrangement, (C6) multiple rows with $s_y = 3d$ and $s_x = 3d$ in uniform arrangement, and (C7) multiple rows with $s_y = 3d$ and $s_x = 3d$ in staggered arrangement.

The flume is 40 m long, 0.8 m wide and 0.8 m high. A continuous inflow of water was pumped into the flume, while the water level upstream from the cylinder array was kept at a constant level of $h =$ 0.55 m. The pumping rates were adjusted to obtain three different depth-averaged flow velocities of 0.1, 0.2 and 0.4 $\mathrm{m s}^{-1}$. These values corresponded with *Re* values of 4000, 8000 and 16,000, where *Re* is the Reynolds number based on cylinder diameter and incoming velocities upstream from the structure. A frame with cylinders was placed in the middle of the flume, as illustrated in Fig. 7a. The physical model consisted of a grid of $0.76 \times {0.76}$ m, where aluminum cylinders could be introduced in different arrangements. The elements were held together by a top and a bottom plate. The tested volumetric porosities ranged between *n* = 0.64 and 0.9. The cylinder diameter was $d =$ 0.04 m for all experiments. The tested configurations are illustrated in Fig. 7b. The properties of the configurations are summarized in Table 1.

The locations of the instruments used during the experiments are presented in Fig. 7a. All the instruments were measuring continuously with a frequency of 100 Hz. An electromagnetic flow meter (EMF) was placed at a distance of 0.4 m upstream from the structure, at a fixed height of 0.4 m from the bottom. The EMF measured with an accuracy of 1%^41^. The instantaneous flow velocities were measured with a Nortek Vectrino acoustic velocimeter (ADV) at a fixed height of 0.4 m from the bottom. The ADV probe was installed 0.04 m upstream from the gap between two elements. The ADV measured the approaching flow before it was accelerated between two elements, and it had an accuracy of approximately 1%^42^. The output of both velocity sensors was in volts, and the velocities were obtained from linear regression, using separate calibration factors for each instrument. The hydrodynamic loads acting on one single cylinder were recorded with a SCAIME load cell mounted on the upper part of the element, measuring in volts with 0.017% accuracy^43^. The load cells were calibrated using known weights, and fitting a linear relationship between weight and voltage output. The forces were calculated by multiplying the sensor output by the calibration factor, and by the acceleration of gravity.

The bulk drag coefficients were determined by using Eq. (1) with the mean force measured at the center of each configuration, and the mean incoming velocity recorded by the EMF. The average forces and velocities were calculated using a moving average over intervals of 20 s.

## Data Availability

The dataset collected by Jansen^29^ is available in the data repository of Delft University of Technology: https://data.4tu.nl/, with the 10.4121/12764780.v1.

## References

[1] Badola R, Hussain S (2005). Valuing ecosystem functions: An empirical study on the storm protection function of Bhitarkanika mangrove ecosystem, India. Environ. Conserv..

[2] Danielsen F (2005). The Asian Tsunami: A protective role for coastal vegetation. Science.

[3] Nguyen T, Parnell KE (2017). Gradual expansion of mangrove areas as an ecological solution for stabilizing a severely eroded mangrove dominated muddy coast. Ecol. Eng..

[4] FAO (2007). The world’s mangroves 1980–2005. FAO Forest. Pap..

[5] Winterwerp J, Erftemeijer P, Suryadiputra N, Van Eijk P, Zhang L (2013). Defining eco-morphodynamic requirements for rehabilitating eroding Mangrove-Mud coasts. Wetlands.

[6] Schmitt K, Albers T, Pham T, Dinh S (2013). Site-specific and integrated adaptation to climate change in the coastal mangrove zone of Soc Trang Province, Viet Nam. J. Coast. Conserv..

[7] van Wesenbeeck BK (2015). Aquaculture induced erosion of tropical coastlines throws coastal communities back into poverty. Ocean Coast. Manag..

[8] Cuong C, Brown S, Huu H, Hockings M (2015). Using Melaleuca fences as soft coastal engineering for mangrove restoration in Kien Giang, Vietnam. Ecol. Eng..

[9] Winterwerp J, Borst W, Vries M (2005). Pilot study on the erosion and rehabilitation of a Mangrove Mud coast pilot study on the erosion and rehabilitation of a Mangrove Mud Coast. J. Coast. Res..

[10] Ecoshape. *Building with Nature Indonesia; Securing Eroding Delta Coastlines. Technical report.* (2015).

[11] Etminan V, Lowe R, Ghisalberti M (2017). A new model for predicting the drag exerted by vegetation canopies. Water Resour. Res..

[12] Zdravkovich M (1986). The effects of interference between circular cylinders in cross flow. J. Fluids Struct..

[13] Sumer B, Fredsoe J (1997). Hydrodynamics Around Cylindrical Structures.

[14] van Rooijen A, Lowe R, Ghisalberti M, Conde-frias M (2018). Predicting current-induced drag in emergent and submerged aquatic vegetation canopies. Front. Mar. Sci..

[15] Ali S, Uijttewaal W (2011). Flow resistance of vegetated oblique weir-like obstacles during high water stages. Hydrol. Earth Syst. Sci. Discuss..

[16] van Houwelingen, J. *et al.* The effect of finger spreading on drag of the hand in human swimming. *J. Biomech.* **63** (2016). arXiv:1611.08578v1. 10.1016/j.jbiomech.2017.08.002 28823502

[17] Gu Z, Sun T (1999). On interference between two circular cylinders in staggered arrangement at high subcritical Reynolds numbers. J. Wind Eng. Ind. Aerodyn..

[18] Liu X, Levitan M, Nikitopoulos D (2008). Wind tunnel tests for mean drag and lift coefficients on multiple circular cylinders arranged in-line. J. Wind Eng. Ind. Aerodyn..

[19] Sumner D (2010). Two circular cylinders in cross-flow: A review. J. Fluids Struct..

[20] Zhou Y, MahbubAlam M (2016). Wake of two interacting circular cylinders: A review. Int. J. Heat Fluid Flow.

[21] Tanino Y, Nepf HM (2008). Laboratory investigation of mean drag in a random array of rigid, emergent cylinders. J. Hydraul. Eng..

[22] Tinoco R, Cowen E (2013). The direct and indirect measurement of boundary stress and drag on individual and complex arrays of elements. Exp. Fluids..

[23] Sonnenwald F, Stovin V, Guymer I (2019). Estimating drag coefficient for arrays of rigid cylinders representing emergent vegetation. J. Hydraul. Res..

[24] Blevins R (2005). Forces on and stability of a cylinder in a wake. J. Offshore Mech. Arct. Eng..

[25] Nepf H (1999). Drag, turbulence, and diffusion in flow through emergent vegetation. Water Resour. Res..

[26] Eames I, Jonsson C, Johnson P (2011). The growth of a cylinder wake in turbulent flow. J. Turbulence.

[27] Meftah B, Mossa M (2017). Prediction of channel flow characteristics through square arrays of emergent cylinders. Phys. Fluids.

[28] Mossa M, Meftah M, De Serio F, Nepf H (2017). How vegetation in flows modifies the turbulent mixing and spreading of jets. Sci. Rep..

[29] Jansen, W. Wave dissipation in a permeable structure. *MSc thesis, Delft University of Technology* (2019).

[30] White FM (1991). Viscous Fluid Flow.

[31] Nezu, I. & Nakagawa, H. *Turbulence in Open-channel Flow*, 7th edn. (IAHR Monograph, Balkema, Rotterdam, The Netherlands, 1993)

[32] Kim S, Stoesser T (2011). Closure modeling and direct simulation of vegetation drag in flow through emergent vegetation. Water Resour. Res..

[33] Petryck, S. *Drag on cylinders in open channel flow*. Ph.D. thesis, Colorado State University, Fort Collins (1969).

[34] Li R, Shen HW (1973). Effect of tall vegetations on flow and sediment. J. Hydraul. Div..

[35] Lindner K. Der (1982). Strömungswiderstand von Pflanzenbeständen. Mitt. Liechtweiß Inst. Wasserbau Tech. Univ. Bräunschweig.

[36] Schoneboom T, Aberle J, Dittrich A (2011). Spatial variability, mean drag forces, and drag coefficients in an array of rigid cylinders. Exp. Methods Hydraul. Res..

[37] Rizzo C, de Barros F (2017). Minimum hydraulic resistance and least resistance path in heterogeneous porous media. Water Resour. Res..

[38] Morison JR, O’Brien MP, Johnson JW, Schaaf SA (1950). The force exerted by surface waves on piles. Pet. Trans..

[39] Keulegan GH, Carpenter LH (1958). Forces on cylinders and plates in an oscillating fluid. J. Res. Nat. Bur. Std..

[40] Gijón Mancheño, A. *et al.* Wave transmission and drag coefficients through dense cylinder arrays: Implications for designing nature-based structures. *Ecol. Eng.* (2021) **(Under review)**.

[41] Hydraulics, D. User’s manual for the Delft hydraulics four quadrant electromagnetic liquid velocity meter (1990).

[42] Nortek. Vectrino technical specification. https://www.nortekgroup.com/export/pdf/Vectrino.pdf (2020).

[43] SCAIME. Load cell-single point—AL. https://scaime.com/product/post/al (2020).

